# Predictive Role of Neutrophil to Lymphocyte Ratio in Adnexal Torsion: A Systematic Review and Meta-Analysis

**DOI:** 10.1155/2022/9680591

**Published:** 2022-11-03

**Authors:** Shokoufeh Khanzadeh, Hossein Tahernia, Jairo Hernandez, Camila Sarcone, Brandon Lucke-Wold, Amirhosseinn Salimi, Fatemeh Tabatabaei

**Affiliations:** ^1^Student Research Committee, Tabriz University of Medical Sciences, Tabriz, Iran; ^2^Ranfanjan University of Medical Science, Ranfanjan, Iran; ^3^Department of Neurosurgery, University of Florida, Gainesville, Florida, USA; ^4^Student Research Committee, Shahid Sadoughi University of Medical Sciences, Yazd, Iran; ^5^Department of Obstetrics and Gynaecology, School of Medicine, Tabriz University of Medical Sciences, Tabriz, Iran; ^6^Department of Gynaecologic Laparoscopic Surgeries, Al-Zahra Hospital, Tabriz University of Medical Sciences, Tabriz, Iran

## Abstract

**Introduction:**

The goal of this systematic review and meta-analysis was to consolidate the available data on the role of the neutrophil to lymphocyte ratio (NLR) in predicting adnexal torsion (AT), to help guide clinical decision-making and outcomes.

**Methods:**

We used Web of Science, PubMed, and Scopus to conduct a systematic search for relevant publications published before June 26, 2022. We reported standardized mean difference (SMD) with a 95% confidence interval (CI). Because a significant level of heterogeneity was found, we used the random-effects model to calculate pooled effects. We used the Quality Assessment of Diagnostic Accuracy Studies 2 (QUADAS-2) too for quality assessment.

**Results:**

Overall, 15 articles were included in the analysis. A random-effects model revealed that patients with AT had elevated levels of NLR compared to those with other adnexal masses (SMD = 1.06, 95%CI = 0.67 to 1.45, *p* < 0.001). So, NLR had diagnostic value. In the subgroup analysis according to ethnicity, we found that Caucasian patients with AT had elevated levels of NLR compared to patients who were operated due to adnexal mass and reported as having a benign ovarian cyst, without torsion (SMD = 1.12, 95%CI = 0.71 to 1.54, *p* < 0.001). However, in the case of East Asian patients, there was no difference between cases and controls (SMD = 0.86, 95%CI = −0.21 to 1.94, *p* = 0.11). The pooled sensitivity of NLR was 0.79 (95%CI = 0.72–0.85), and the pooled specificity was 0.84 (95% CI, 0.74–0.91).

**Conclusion:**

In conclusion, there has been an interest in the use of NLR as a diagnostic marker for AT.

## 1. Introduction

Neutrophil to lymphocyte ratio (NLR) has been studied in the context of gynecological and obstetrical diseases, serving as a positive marker for cervical cancer, endometrial cancer, and uterine sarcomas [1]. Recent data has highlighted the importance of NLR as a marker for adnexal torsion (AT) diagnosis [2]. AT is an emergent gynecological phenomenon which, without early intervention and an accurate diagnosis, could result in ovarian loss, infertility, and death. It is defined as the partial or complete twisting of the suspensory ligament that supports the blood supply to the ovary [3]. The nonspecific symptoms of AT, such as vomiting, nausea, and low-grade fever, often complicates its diagnosis. As studies continue to emerge on the utility of NLR in AT [1–15], the need for a systematic review to guide clinical decision-making is essential. The key is to understand what an elevated ratio might mean for a patient with possible AT, to institute early interventions and improve outcomes. To the best of our knowledge, there are no systematic reviews in the current literature regarding NLR in this context. The goal of this systematic review and meta-analysis was to consolidate the available data on the role of the NLR in predicting AT, to help guide clinical decision-making and outcomes.

## 2. Methods

### 2.1. Eligibility Criteria

We included human studies according to the following eligibility criteria, based on PICO [16]:

- Population. Patients undergoing surgery for adnexal mass
- Intervention/exposure. All types of AT
- Control. Patients who were operated due to adnexal mass and reported as having a benign ovarian cyst, without torsion or rupture
- Outcomes. NLR level
- Study design. We included case-control or cross-sectional studies. However, we did not limit our search to any particular research design

### 2.2. Search Strategy and Study Selection

We performed a comprehensive literature search in the databases of PubMed, Web of Science, and Scopus, from inception until June 26, 2022, by applying the following search strategy: ((“neutrophil”[All Fields] AND “lymphocyte”[All Fields] AND “ratio”[All Fields]) OR “Neutrophil-to-lymphocyte ratio”[All Fields] OR “NLR”[All Fields]) AND ((“adnexal”[All Fields] OR “ovarian”[All Fields] OR “fallopian”[All Fields]) AND “torsion”[All Fields]).

Two authors independently screened abstracts. Full text of relative papers was retrieved. We also investigated the references of relevant review or original articles in order to identify further eligible studies. A third person resolved disagreements between the two authors who screened the papers.

### 2.3. Data Extract and Quality Assessment

We extracted the following data: the first author, year of publication, study location, study design, the number of cases and controls separately, mean ± SD of NLR level in cases and controls, or sufficient data for estimating the mean ± SD such as median and interquartile range (IQR) or/and range. Two reviewers assessed the quality of included studies independently using the validated Quality Assessment of Diagnostic Accuracy Studies 2 (QUADAS-2) tool.

### 2.4. Statistical Analysis

Standardized mean differences (SMD), and 95% confidence intervals (CIs), were applied to report forest plots of continuous data. We considered *p* < 0.05 as statistically significant. If a study did not report mean ± SD, we estimated them from the median and range [17]. We used *Q* statistic (significance level at *p* < 0.10) to test the heterogeneity of SMD across included articles. Additionally, we calculated the *I*^2^ statistic as a quantitative measurement to evaluate inconsistency across studies (*I*^2^ < 25%, no heterogeneity; *I*^2^ between 25% and 50%, moderate heterogeneity; *I*^2^ between 50% and 75%, large heterogeneity; and *I*^2^ > 75%, extreme heterogeneity). Because of high heterogeneity, we applied a random-effects model to report the pooled SMD and corresponding 95% confidence intervals.

We used Egger test to assess the potential publication bias (at the *p* < 0.05 level of significance). In order to evaluate the diagnostic value of NLR in AT, we used “metandi” command to report summary receiver-operating characteristic (SROC) curve, the sensitivity, specificity, diagnostic odds ratio (DOR), negative likelihood ratio, and positive likelihood ratio. We used Stata 14 (STATA Corp., College Station, TX, USA) for the statistical analyses. The current study completely followed the PRISMA statement about the reporting of systematic reviews and meta-analyses [18] and the broader EQUATOR guidelines [19].

## 3. Results

### 3.1. Search Results and Included Studies

**Figure 1:**
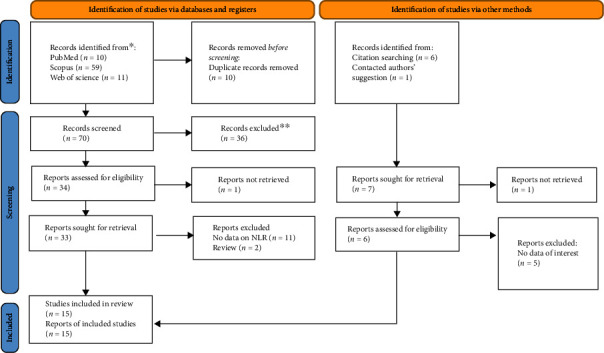
PRISMA 2020 flow diagram for new systematic reviews which includes searches of databases, registers, and other sources.

The database search and manual search of the article citation list yielded a total of 87 results. Finally, 15 papers were included in this systematic review and meta-analysis after duplicates and nonrelevant records were removed.  Figure 1 shows the PRISMA flow diagram, indicating the process of inclusion and exclusion in details.

### 3.2. Characteristics of the Included Studies

In total, 15 articles were included in the analysis [1–15], including 1156 patients with AT and 1460 patients with other adnexal masses. All of them were retrospective [1–15]. One study was written in Turkish [4] and 14 in English [1, 2, 3, 5–15]. One of them was conference paper [8], and 14 were journal papers [1–7, 9–15]. Ten studies were conducted in Turkey [1–5, 10–13, 15], three in Korea [6–8], one in Israel [9], and one in China [14]. Ten studies conducted receiver-operating characteristic (ROC) curve analysis [1–4, 6, 7, 10, 13–15].  Table 1 shows the overall characteristics of the included articles.

### 3.3. NLR Level in Patients with AT

**Figure 2:**
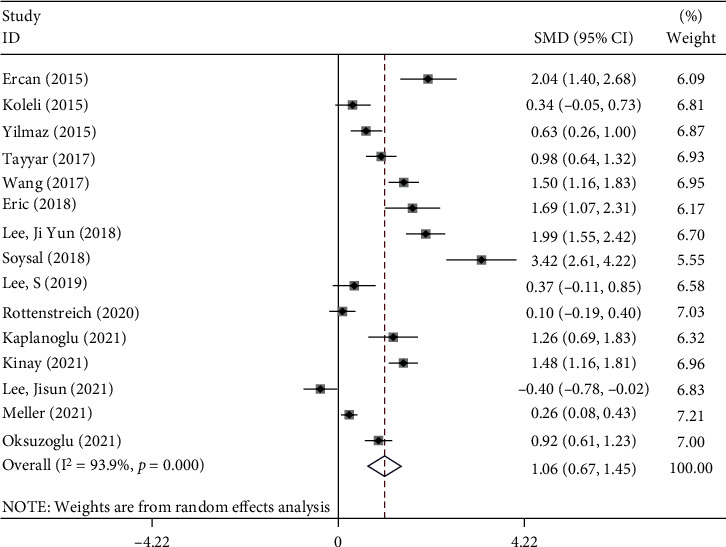
Meta-analysis of differences in NLR level between patients with AT compared to patients who were operated due to adnexal mass and reported as having a benign ovarian cyst, without torsion.

A random-effects model revealed that patients with AT had elevated levels of NLR compared to patients who were operated due to adnexal mass and reported as having a benign ovarian cyst, without torsion (SMD = 1.06, 95%CI = 0.67 to 1.45, *p* < 0.001, Figure 2). So, NLR had diagnostic value.

**Figure 3:**
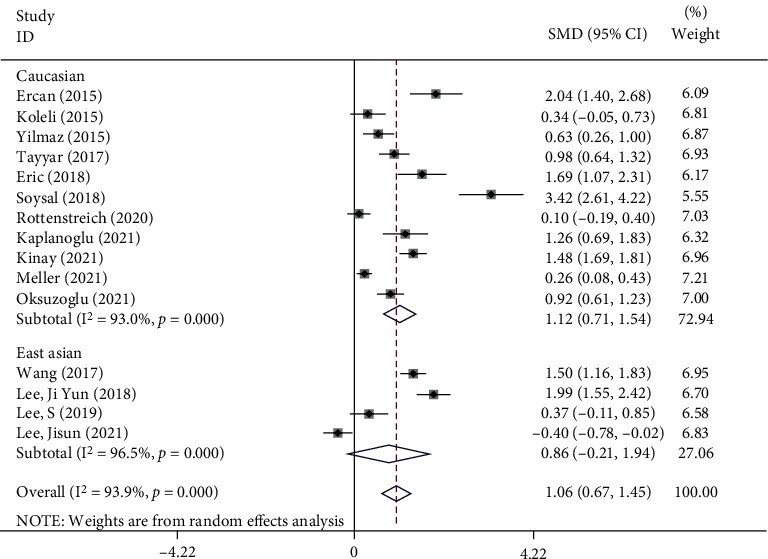
Subgroup analysis of differences in NLR level between patients with AT compared to patients who were operated due to adnexal mass and reported as having a benign ovarian cyst, without torsion, according to ethnicity.

In the subgroup analysis according to ethnicity, we found that Caucasian patients with AT had elevated levels of NLR compared to patients who were operated due to adnexal mass and reported as having a benign ovarian cyst, without torsion (SMD = 1.12, 95%CI = 0.71 to 1.54, *p* < 0.001). However, in the case of East Asian patients, there was no difference between cases and controls (SMD = 0.86, 95%CI = −0.21 to 1.94, *p* = 0.11) (Figure 3).

### 3.4. Diagnostic Value of NLR

**Figure 4:**
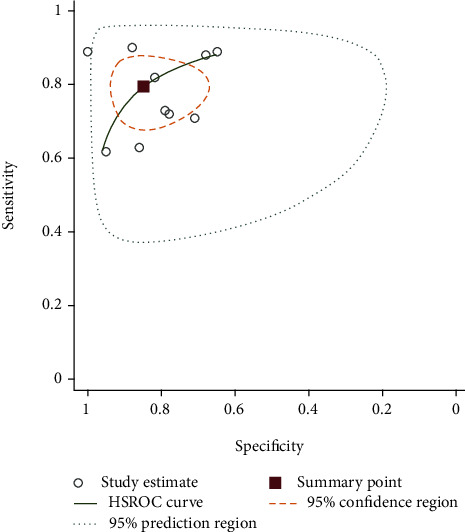
SROC curve of included studies assessing diagnostic value of NLR for AT.

The pooled sensitivity of ten studies was 0.79 (95%CI = 0.72–0.85), and the pooled specificity was 0.84 (95% CI, 0.74–0.91). The pooled positive likelihood ratio, negative likelihood ratio, and DOR of NLR were 5.24 (95%CI = 2.96–9.26), 0.24 (95%CI = 0.17–0.33), and 21.71 (95%CI = 9.96–47.28), respectively (Figure 4).

### 3.5. Publication Bias and Study Quality

**Figure 5:**
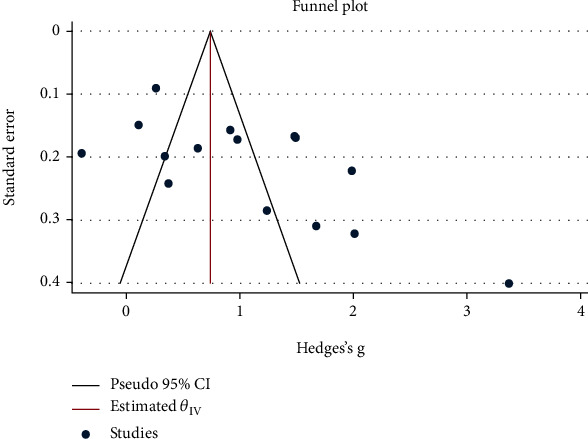
Funnel plot assessing publication bias.

**Figure 6:**
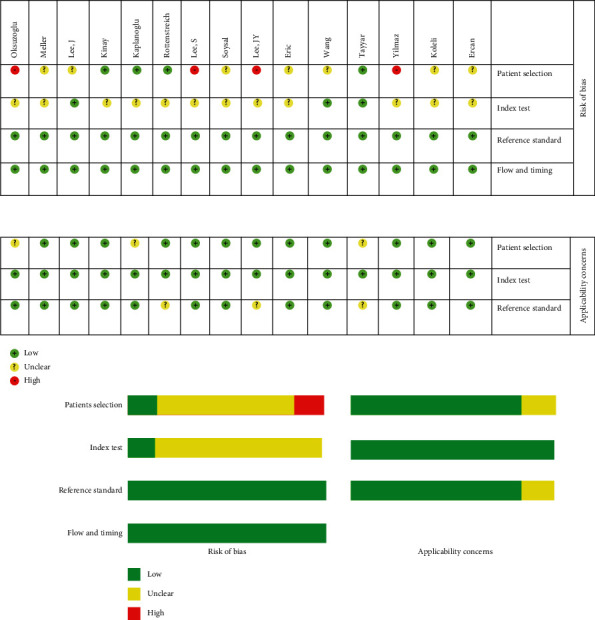
Quality assessment of included studies using QUADAS-2 tool.

As seen in Figure 5, there was not any publication bias among studies on the usefulness of NLR for the prediction of AT (Begg test's *p* = 0.06). In addition, the methodological quality of the included studies is presented in Figure 6.

## 4. Discussion

Our study had two main findings. First, the NLR level was significantly elevated in patients with AT compared to patients who were operated due to adnexal mass and reported as having a benign ovarian cyst, without torsion. Second, Caucasian patients with AT had a higher level of NLR compared to patients who were operated due to adnexal mass and reported as having a benign ovarian cyst, without torsion, but NLR in East Asian patients showed no diagnostic utility. It is important to note the dynamic roles of neutrophils and lymphocytes in the setting of AT to understand the significance of their relative proportion. In patient with AT, an increase in neutrophil count and decrease in lymphocyte count has been observed compared to controls, resulting in a higher NLR [1]. Acute ischemia, induced by AT, produces an inflammatory response maintained by neutrophils via the release of proinflammatory cytokines. The cytokines released include IL-2, IL-6, IL-1*β*, and TNF-*α* which are produced during ischemic and reperfusion injury [20]. The ischemic state also induces the release of endogenous cortisol, which consequently leads to lymphopenia [21].

Recent evidence demonstrates that elevated NLR is a useful parameter in the diagnosis of AT. Tas et al. [22] performed a retrospective, case-control study of 296 subjects comparing preoperative blood count parameters in AT vs. control patients. NLR, platelet-to-lymphocyte ratio (PLR), and mean white blood cell count (WBC) were significantly higher in the cases compared to controls (*p* < 0.05). The addition of mean platelet volume could improve the accuracy of NLR results since there was an independent association between a low mean platelet volume and AT (*p* < 0.05). Nissen et al. [21] retrospectively investigated 88 pediatric patients (3 days up to 17.8 years) with ovarian pathology who were assigned to the AT or control group. Similarly, NLR, PLR, and WBC were all significantly elevated in patients aged older than 1 year. These findings might not be specific to female gonads, as a meta-analysis by Zhu et al. [23] displayed elevated NLR in testicular torsion patients.

Interestingly, we found a difference in NLR predictive value for AT when comparing studies with Caucasian patients compared to East Asian patients (*p* = 0.11). Specifically, higher preoperative NLR values were found in Caucasian patients with AT relative to those with other adnexal masses, but this effect was not replicated in the East Asian group. We propose that these differences may be caused by differences in diet, which leads to differences in microbiome composition [24]. Microbiomes vary substantially by ethnicity, and ethnicity is a reliable proxy for dietary and lifestyle variation between groups [25, 26]. The relevance of the microbiome and diet has been shown in other inflammatory states that display elevated NLR, like anastomotic leakage and peritonitis, in which modulations of diet/microbiome impact the outcomes of the group [27–29]. Experiments manipulating diet/microbiome in animal models with AT are needed to directly address our findings, but the limited literature suggests that the differences between Caucasian and East Asian individuals in our study may be partially attributed to dietary and lifestyle variation between ethnicities.

Ultrasonography is generally used to make the diagnosis of AT. Findings of fluid in the pelvic cavity, abnormal positioning of the ovary, and adnexal cysts are often present in patients with AT [12]. Yet, in approximately half of pediatric AT cases, the ultrasonography was found to be normal, indicating the need for a more specific diagnostic method [30]. Color Doppler ultrasound is also widely used in the diagnosis of AT, where a lack or decrease in blood flow could yield an AT diagnosis. However, in 60% of cases with AT, blood flow was found to be normal due to the dual blood supply from ovarian and uterine arteries [31]. Therefore, the use of color Doppler ultrasound is debatable. White blood cell count (WBC) is often examined in inflammatory cases, such as in AT. When compared to NLR, the specificity and sensitivity of WBC were lower in patients with AT [2]. Kinay et al. reported that only 55.2% of women with a diagnosis of AT had an elevated WBC count. These results suggest that NLR might precede WBC elevation and could be useful to diagnose AT in women with normal WBC counts, further supporting the use of NLR in diagnosis of AT. Kinay et al. reported that with an NLR cut-off of 2.51, a sensitivity of 72% and a specificity of 78% were observed in AT cases. In addition to the previously mentioned diagnostic tools, the use of platelet-to-lymphocyte ratio has been investigated. However, its sensitivity and specificity were found to be lower than NLR in ROC analyses [2].

Due to the nonspecific signs and symptoms of AT, additional imaging modalities, such as computed tomography (CT) and magnetic resonance imaging (MRI), are required to differentially diagnose AT from other abdominal pathologies. Mandoul et al. [32] reported a sensitivity of 97% and specificity of 81% for the use of CT in the diagnosis of AT. With respect to MRI, a sensitivity of 100% and specificity of 77.8% were observed [33]. While these imaging modalities have a higher sensitivity and specificity than NLR, lack of affordability and availability favors other imagining techniques for the diagnosis of AT. Color Doppler ultrasound is widely available and affordable. However, a sensitivity of 43.8% and specificity of 91.7% were observed [34]. When compared to ultrasound, NLR had a higher sensitivity (72%) but lower specificity (78%) for the diagnosis of AT [2]. In comparison to the current diagnostic methods, NLR offers a rapid, affordable, and reliable tool for the diagnosis of AT. Some studies were included solely in the qualitative review of our study. All of them assessed the relationship between NLR and AT and reported that NLR could predict AT with impressive sensitivity/specificity, similar to our findings [21–23].

Reducing ischemic time is essential in the prevention and reduction of ovarian tissue damage. The most effective clinical approach is early conservation surgery or detorsion. However, this treatment is often proceeded by neutrophil infiltration, an increase in free oxygen radicals and cytokines [35]. The combination of neutrophilia and lymphopenia results in the increased NLR observed in AT. Inflammation, therefore, is important in mediating the severity of ischemia and reperfusion in AT. Anti-inflammatory and anti-antioxidant drugs have been investigated in their role of treating AT. In rats with ovarian torsion who underwent ischemic and ischemic-reperfusion damage, an increase in TNF-*α* expression was observed in antral follicular cells, inflammatory cells, and endothelial cells [35]. Rosmarinic acid, a naturally occurring antioxidant with anti-inflammatory properties, was given to the ischemic and ischemic-reperfusion groups. This resulted in a decrease in inflammation-induced TNF-*α* expression, indicating the beneficial effect of this drug on preventing ischemic damage after ischemia/reperfusion [35].

Urapidil, a vasodilator drug, was found to decrease IL-1*β* and TNF-*α* levels, and consequently, inflammation, in a dose-dependent manner in rats with ovarian torsion [20]. Nicorandil, a KATP opener and nitrate agonist, showed a negative immune-expression of COX-2 and downregulation of proinflammatory cytokines, supporting its role as an anti-inflammatory drug [36]. Urapidil and Nicorandil's anti-inflammatory and antioxidant properties had a protective effect in ovarian torsion in rats and allowed for the preservation of ovarian reserve [20, 36]. In another study, etoricoxib, a selective COX-2 inhibitor, was found to prevent the inflammatory response and production of reactive oxygen species causes by ischemia/reperfusion in rat ovarian tissue [37]. The positive outcomes from the use of anti-inflammatory and antioxidant drugs in the treatment of ischemia and reperfusion injury in animal models with ovarian torsion aid in understanding the relationship to NLR.

Several limitations should be considered when interpreting our results. First, all of the included studies were retrospective; second, the majority of the included studies were conducted only one country; third, heterogeneity among the studies which may reflect differences in age, ethnicity, type of adnexal mass of control group, and time of blood collection.

In conclusion, there has been an interest in the use of NLR as a diagnostic marker for AT. In our review, data collected from available studies demonstrate a variable degree of support for the diagnostic potential of NLR in this context. However, in general, our meta-analysis suggests that NLR has significant diagnostic potential for AT. This predictive potential increases even further when combined with other diagnostic tools such as mean platelet volume.

## Figures and Tables

**Table 1:** General characteristics of included studies.

First author	Year	Country	Design	Ethnicity	NLR cut-off	SEN	SP	AT group		Control group	
								*N*	NLR	*N*	NLR
Ercan [1]	2015	Turkey	Retrospective	Caucasian	3.0	89%	100%	27	6.44 ± 2.92	30	2.24 ± 0.63
Koleli [5]	2015	Turkey	Retrospective	Caucasian	_	_	_	51	0.75 ± 0.32	50	0.64 ± 0.33
Yilmaz [15]	2015	Turkey	Retrospective	Caucasian	2.44	71%	71%	44	4.30 ± 2.60	92	2.70 ± 2.50
Tayyar [13]	2017	Turkey	Retrospective	Caucasian	2.9	73%	79%	72	6.30 ± 4.60	77	2.80 ± 2.20
Wang [14]	2017	China	Retrospective	East Asian	3.56	62%	95%	68	5.28 ± 3.33	120	2.15 ± 0.77
Eric [4]	2018	Turkey	Retrospective	Caucasian	2.77	90%	88%	20	7.27 ± 5.12	40	2.17 ± 0.85
Lee, JY [7]	2018	South Korea	Retrospective	East Asian	3.35	63%	86%	24	6.20 ± 4.90	360	2.30 ± 1.60
Soysal [12]	2018	Turkey	Retrospective	Caucasian	_	_	_	35	8.52 ± 2.40	25	2.22 ± 0.22
Lee, S [8]	2019	South Korea	Retrospective	East Asian	_	_	_	29	9.48 ± 6.60	41	7.41 ± 4.73
Rottenstreich [11]	2020	Turkey	Retrospective	Caucasian	_	_	_	143	5.00 ± 4.10	65	4.60 ± 3.20
Kaplanoglu [3]	2021	Turkey	Retrospective	Caucasian	2.45	82%	82%	28	7.13 ± 5.89	29	1.91 ± 0.69
Kinay [2]	2021	Turkey	Retrospective	Caucasian	2.45	72%	78%	67	5.90 ± 4.30	134	2.10 ± 0.80
Lee, J [6]	2021	South Korea	Retrospective	East Asian	2.80	89%	65%	37	6.06 ± 3.19	95	8.04 ± 5.50
Meller [9]	2021	Israel	Retrospective	Caucasian	_	_	_	445	4.20 ± 3.49	170	3.30 ± 3.50
Oksuzoglu [10]	2021	Turkey	Retrospective	Caucasian	2.68	88%	68%	66	6.50 ± 3.70	132	3.40 ± 3.20

*N*: number; NLR: neutrophil to lymphocyte ratio; NOS: the Newcastle-Ottawa Quality Assessment Scale; AT: adnexal torsion; SEN: sensitivity; SP: specificity.

## Data Availability

The dataset supporting the conclusions of this article is included within the article.
